# The similarity and variability of the iridoid glycoside profile and antioxidant capacity of aerial and underground parts of *Lamiophlomis rotata* according to UPLC-TOF-MS and multivariate analyses†

**DOI:** 10.1039/c7ra10143k

**Published:** 2018-01-10

**Authors:** Dan Zhang, Yun-ling Gao, Sheng Jiang, Yiwen Chen, Yi Zhang, Zheng Pan

**Affiliations:** College of Traditional Chinese Medicine, Chongqing Medical University Chongqing China letter2013@sina.com; School of Bio-information, Chongqing University of Post and Telecommunications Chongqing China; Chongqing Institute of Food and Drug Control Chongqing China; College of Ethnic Medicine, Chengdu University of Traditional Chinese Medicine Chengdu China

## Abstract

*Lamiophlomis rotata* (*L. rotata*) is a Tibetan medicinal herb used for centuries that contains iridoid glycosides (IGs), which are pharmacologically active ingredients and can be used for quality control. The IG profiles of the underground and aerial parts of the plant were determined by UPLC-TOF-MS to evaluate the similarity and variability of the different herbal parts listed in the Chinese Pharmacopoeia. Twenty-six IGs were detected in the total ion current (TIC) profile of *L. rotata*, and twenty-two of these were identified by comparing the retention times and mass spectra of the compounds to those of authentic standards. Among these compounds, five IGs with the same molecular formula (C_17_H_26_O_11_) were identified for the first time by mass spectrometry based on their different hydroxyl group-substituted positions. The aerial part has a similar chemical profile to that of the roots. The difference between the two parts was determined by multivariate statistical analysis of the UPLC-TOF-MS data of 24 specimens. Sesamoside was explored as the most characteristic marker to distinguish the two parts of *L. rotata*. To further estimate the distinction between the two parts, the content of total IGs and the antioxidant capacity were investigated in samples from different locations. The aerial parts showed a high content of total IGs and high antioxidant capacity, although not higher than those of the roots. The results also suggest the dosage should be increased when the aerial parts are used as crude medicinal materials instead of the underground parts.

## Abstract 2

*Lamiophlomis rotata* (*L. rotata*) is a Tibetan medicinal herb used for centuries that contains iridoid glycosides (IGs), which are pharmacologically active ingredients and can be used for quality control.

## Graphical abstract

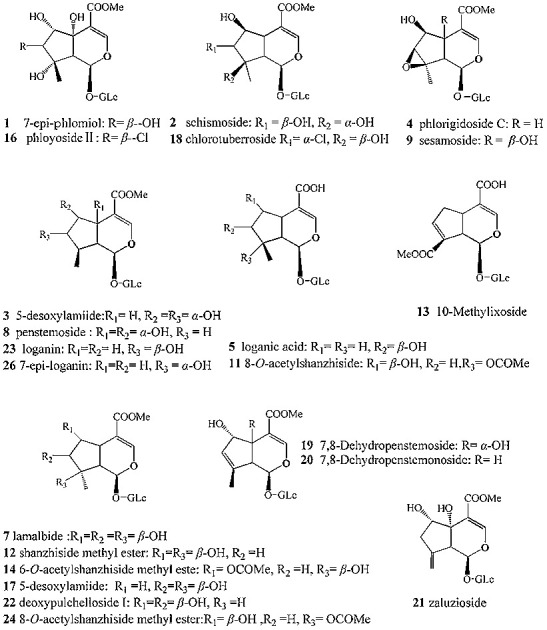

## 1. Introduction

*Lamiophlomis rotata* (Benth.) Kudo (*L. rotata*) is a folk medicinal plant that is widespread throughout the Gansu, Sichuan, Qinghai and Tibet provinces in China.^1^ It has been traditionally used for the treatment of knife and gun wounds and to promote haemostasis for many centuries.^2^ In 1995, preparations made from the root of *L. rotata* were officially listed in the Chinese Pharmacopoeia (Chin. Ph. 1995 edition) for the first time.^3^ In the Chin. Ph. 2005 edition, the whole plant was included.^4^ Finally, the aerial parts of *L. rotata* have been recorded as a raw material in the Chin. Ph. 2010 & 2015 editions.^5,6^

The constituents of *L. rotata* include various iridoid glycosides, phenylpropanoids, flavonoids, *etc.* Among different types of compounds, luteolin and total flavonoids have been used for quality control. However, there is little evidence suggesting that phenylpropanoids and flavonoids are responsible for the haemostatic activities of the herb, on the contrary, more and more new results proved that iridoid glycosides (IGs) are the major constituents of *L. rotata*, and might contribute to its pharmacological and therapeutic activities. Studies have shown that the IGs in *L. rotata* exhibit analgesic,^7,8^ anti-inflammatory,^9^ and haemostatic bioactivities.^10,11^ Additionally, IGs possess antioxidant activity, which protects against free radical damage in the progression of degenerative diseases such as cardiovascular diseases and ischemic brain injury.^12,13^ Consequently, in parallel with the pharmacological and therapeutic results, the marker compound used for quality control changed from “luteolin” to “shanzhiside methyl ester and 8-*O*-acetyl shanzhiside ethyl ester”, and these two compounds were identified as the principle effective IGs.^5,6^ However, a few of these studies have sought to determine the antioxidant capacity of total IGs in different parts of *L. rotata* after the change in the listing of its medicinal parts. Therefore, comparing the iridoid glycosides constituents between the aerial and underground parts of *L. rotate* is important for their quality control, and for the rationality of the herb parts alteration in the Chinese Pharmacopoeia.

**Fig. 1:**
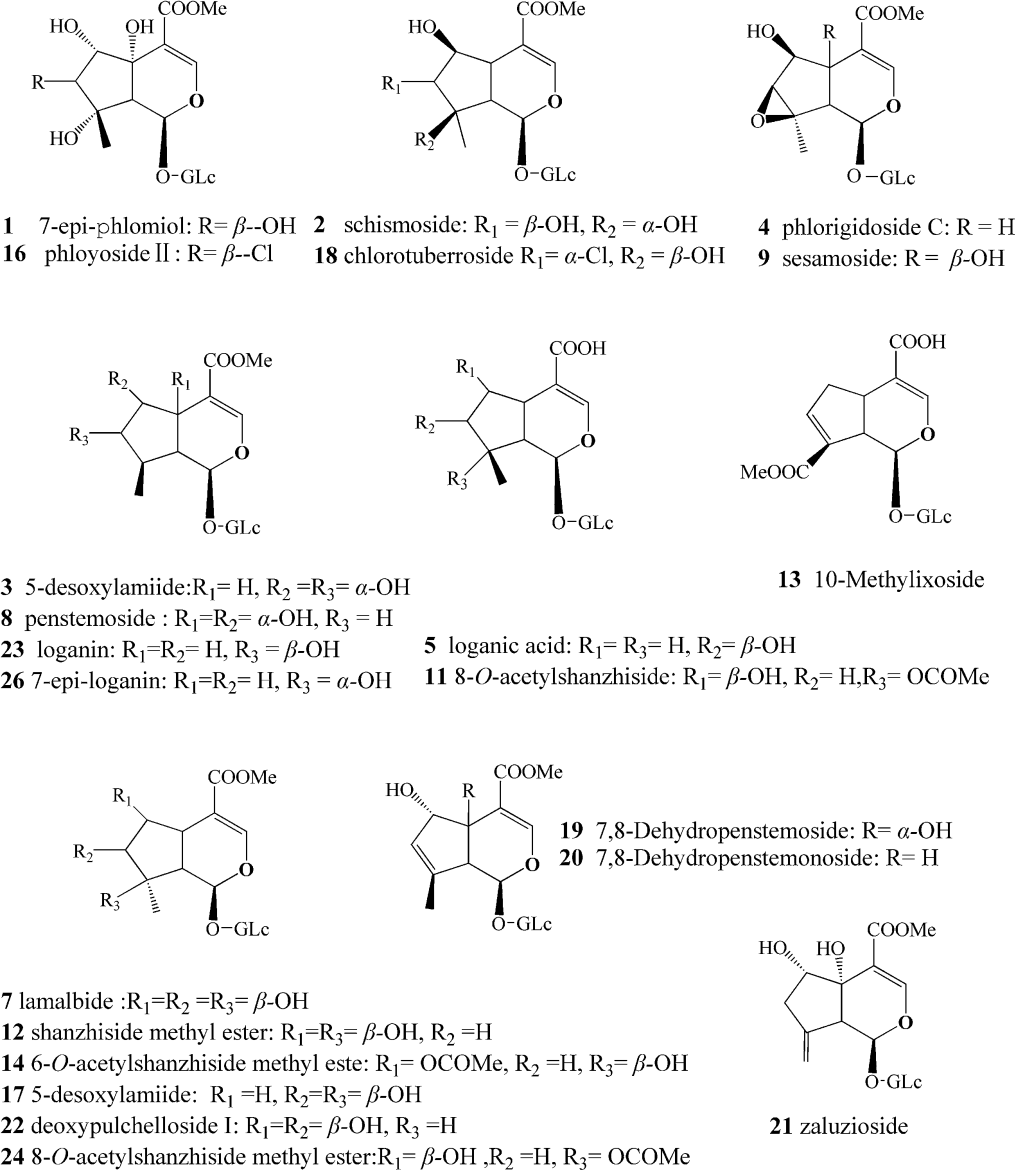
Chemical structures of major components identified from *L. rotata*. The compound numbers represent the same meanings as in Table 2.

More than 20 IGs have been identified in *L. rotate* (Fig. 1),^14–16^ and methods have been established for determining IGs. The levels of IGs in the roots,^17^ in the aerial parts,^18^ and in the whole plants^19^ have been determined. Although some analytical methods have shown that shanzhiside methyl ester and 8-*O*-acetyl-shanzhiside ethyl ester exist in both the aerial and underground parts in *L. rotata*, it must be noted that these methods featuring the quantification of only a few IGs are not comprehensive IG profile analyses, and caution should also be exercised in assuming the similarity of IG composition in the aerial and underground parts of *L. rotata* from various geographical regions. There are very few published accounts on the similarity or variability of IG constituents in the two parts of *L. rotata* originating from different locations.

The authors of this paper have been interested in the metabolite profiling of phytomedicine to standardize botanical products or herbal medicines for their quality and safety.^20–23^ Here, twelve batches of *L. rotata*, including both the aerial and the root parts, were collected from different habitats in China. A UPLC-TOF-MS-based metabolomic approach was employed, combined with principal component analysis (PCA) and orthogonal partial least-squares discriminant analysis (OPLS-DA), to explore the IG distributions in the aerial and underground parts of *L. rotata* in four geographical habitats. To further evaluate the similarity and variability of the two parts of *L. rotata*, the antioxidant properties of the total IGs in different parts of *L. rotata* were investigated by analysing their 1,1-diphenyl-2-picrylhydrazyl free radical (DPPH˙) scavenging activities. This information along with the knowledge obtained regarding the antioxidant properties of the total IGs in different parts of *L. rotata* may help to confirm the alteration of the medicinal parts.

## 2. Materials and methods

### 2.1. Chemicals, reagents, and plant materials

Shanzhiside methyl ester, 8-*O*-acetylshanzhiside methyl ester, sesamoside, 7,8-dehydropenstemoside, phlorigidoside C, 7-*epi*-phlomiol, chlorotuberoside, loganin, and phloyoside II were purified from *L. rotata* in our laboratory and identified by direct comparison of their ^1^H-NMR and ^13^C-NMR spectra to those in the literature;^15–17^ all purities were >95% by HPLC. 1,1-Diphenyl-2-picrylhydrazyl (DPPH), and butylated hydroxytoluene (BHT) were purchased from Aladdin Co. (California, USA). Trichloroacetic acid, ferric chloride potassium ferricyanide and other chemicals used were of analytical grade and were purchased from Sinopharm Chemical Reagent Co., Ltd. (Shanghai, China).

HPLC-grade methanol and formic acid were purchased from Merck (Darmstadt, Germany) and Tedia (Fairfield, OH, USA). Deionized water was prepared using a Millipore water treatment system (Bedford, MA, USA). All other reagents were of analytical grade.

Twelve batches of *L. rotata*, including both the aerial and root parts, were collected from different habitats in Tibet and the Qinghai, Sichuan, and Gansu provinces of China (Table 1). The herbs were authenticated by Professor Yi Zhang (Chengdu University of Traditional Chinese Medicine, Chengdu, China). The samples were carefully divided into aerial and root parts and dried in the shade; voucher specimens were deposited at the College of Ethnic Medicine (Chengdu University of Traditional Chinese Medicine, Chengdu, China) and Chongqing Academy of Chinese Materia Medica (Chongqing, China).

**Table 1:** Populations of *Lamiophlomis rotata* from four provinces used in this paper

Location	Longitude (E)	Latitude (N)	Altitude (m)	No. of samples
Gansu	104.7733–101.9308	33.9361–33.7805	3789–3500	3
Qinghai	101.7353–96.6487	34.5261–31.1594	4178–3519	3
Sichuan	100.5236–98.7732	31.6583–31.4244	4323–3865	3
Tibet	94.2936–90.5775	32.0844–30.0183	4803–4235	3

### 2.2. Sample preparation

Dried samples (1.0 g of powder each) were extracted into 10 ml of 70% (v/v) aqueous methanol in an ultrasonic bath for 30 min and cooled at room temperature. The extraction was repeated three times using fresh aliquots of the solvent. After combining the three aliquots, the solutions were centrifuged at 12 000 rpm for 10 min and filtered through 0.22 μm pore membranes prior to UPLC-TOF-MS analysis. The extract was evaporated to dryness, and the residue was dissolved with ultrapure water and purified using polyamide resin (0.5 g), Samples of suitable concentration (0.1 g ml^−1^) were stored at −4 °C for further analysis.

### 2.3. UPLC-TOF-MS conditions

Analyses were performed on a Waters Acquity UPLC system (Waters, Milford, MA, USA) equipped with a binary solvent delivery system, an auto-sampler, and a photodiode-array detection (DAD) system. The column was a Waters Acquity UPLC BEH C18 column (100 mm × 2.1 mm, 1.8 μm). The mobile phases were (A) water with 0.1% (v/v) formic acid and (B) methanol with 0.1% (v/v) formic acid. The optimized elution conditions were as follows: isocratic 8% (v/v) B (0–1 min); a linear gradient from 8% to 13% B (all v/v) (1–5 min), 13% to 15% B (5–10 min), 15% to 21% B (10–13 min), 21% to 33% B (13–16 min), 33% to 45% B (16–21 min), 45% to 55% B (21–25 min), 55% to 100% B (25–26 min), isocratic 100% B for 1 min, and then back to 8% (v/v) B in 1 min. The flow rate was 0.3 ml min^−1^. The column temperature was 35 °C. The injection volume was 2 μL.

Mass spectrometry data were obtained using a Xevo® G2 Q/TOF (Waters MS Technologies, Manchester, UK) fitted with an ESI† source and controlled by the MassLynx software (ver. 4.1). Both MS and MS^E^ data scans were recorded. MS full scans were acquired in positive ion mode over the range (*m*/*z*) 100–1000 Da in two channels with a scan time of 1 s. The capillary voltages were set to 2500 V (positive mode) and the cone voltage to 40 V.^24^ Nitrogen gas was used both as a nebulizer and for desolvation. The desolvation and cone gas flow rates were 650 and 50 L h^−1^, respectively. The desolvation temperature was 350 °C, and the source temperature was 105 °C. A solution of leucine enkephalin (1 μg ml^−1^) in acetonitrile/water (1 : 1) with 0.1% (v/v) formic acid, delivered at a flow rate of 10 μL min^−1^, served as the lock mass solution; the *m*/*z* value was 556.2771 in the positive mode.

### 2.4. Data processing and statistical analysis

All UPLC-TOF-MS data were analysed using the MarkerLynx software (Waters, Manchester, UK) to identify the IGs in *L. rotata*. The parameters were as follows: retention time 1.5–28 min, mass range 100–1000 Da, retention time tolerance 0.01 min, and mass tolerance 0.01 Da. Peak integration was calculated using the peak width at 5% height (1 s), peak-to-peak baseline noise (0.1), and peak intensity threshold (10). No specific mass or adduct was excluded. For data analysis, a list of the intensities of detected peaks was generated using the retention time (*t*_R_) and the mass data (*m*/*z*) pairs to identify each peak. An arbitrary ID was assigned to each *t*_R_–*m*/*z* pair in the order of their UPLC elution to facilitate data alignment. This procedure was repeated for each run. Ions from different samples were considered to be identical when they had the same *t*_R_ (tolerance within 0.01 min) and *m*/*z* (tolerance within 0.01 Da). If a peak was not detected in a particular sample, that ion intensity was recorded as zero. Sample and ion intensity data were analysed using the PCA and OPLS-DA subroutines of the SIMCA-P multivariate data analysis software (ver. 13.0; Umetrics, Umeå, Sweden).

### 2.5. Determination of total IGs

The content of total IGs was determined according to the first derivative spectrophotometry. This method has been applied in many fields.^25^ Briefly, full-wavelength scans of the samples were performed by a UV-Vis spectrophotometer (UV-1800), and the first derivative was determined to distinctively reflect the relationship of the absorbance of IGs to wavelength. The standard curve was plotted based on shanzhiside methyl ester, and the total IGs content was expressed as mg shanzhiside methyl ester equivalent per g dry weight.

### 2.6. Antioxidant activity

#### 2.6.1. DPPH assay

The antioxidant capacity was studied through evaluation of the free radical scavenging effect on the DPPH radical. For this purpose, 0.8 ml of extract was mixed with 0.1 ml of DPPH solution in methanol.^26^ Ultrapure water was used as a blank control, and various concentrations of BHT were measured as positive controls. After incubation in the dark for 60 min, the absorbance of each mixture was measured at 517 nm using an ultraviolet spectrophotometer. Each test was repeated three times using the same procedure. The radical scavenging activity was expressed as the IC_50_ value in terms of the inhibition ratio.

#### 2.6.2. Reducing power assay

The reducing power was determined based on the reducing capacity of Fe^3+^.^27^ A series of extract concentrations were mixed with potassium ferricyanide (1%, w/v). After the mixture was incubated at 50 °C for 20 min, 10% trichloroacetic acid was added and the mixture allowed to stand for another 10 min. Ultrapure water and 0.1% FeCl_3_ were added last and after this mixture stood for 10 min, the absorbance was read at 700 nm against BHT. Each test was repeated three times. The reducing power of the different extracts was compared by their IC_50_ values in terms of absorbance.

## 3. Results and discussion

### 3.1. IGs identified by UPLC-TOF-MS spectra in aerial and underground parts of *L. rotata*

**Fig. 2:**
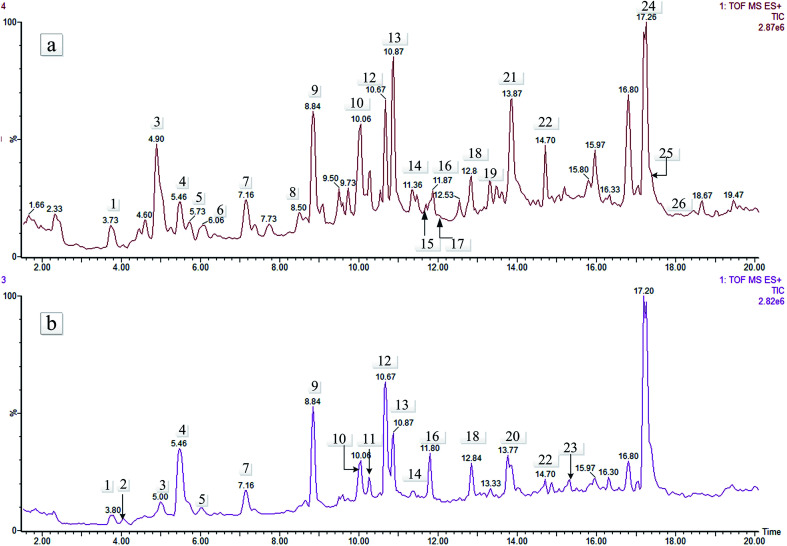
TIC chromatography (positive) of the aerial and root parts of *L. rotata*. Meanings as in Table 1.

Crude extracts of the aerial and underground parts of *L. rotata* were analysed by MS and MS^n^ in positive ion mode. In total, 26 IGs were detected in the total ion current (TIC) profile of *L. rotata* (Fig. 2). To identify the IGs, PubChem, SciFinder Scholar of the American Chemical Society, and the Chinese National Knowledge Infrastructure (CNKI) database of Tsinghua University were searched for the spectral data of IGs reported previously in the *L. rotata* and *Lamium* species, and the results were summarized in a spreadsheet (Excel, Microsoft, WA, USA). Twenty-six IGs were detected in the total ion current (TIC) profile of *L. rotata*, and 22 of these were identified by comparing the retention times and mass spectra of the compounds to those of authentic standards (Table 2). Among these compounds, five IGs with the same molecular formula of C_17_H_26_O_11_ were identified for the first time because of their different hydroxyl group-substituted positions. Additionally, 10-methylixoside, 5-deoxypulchelloside I, 8-*O*-acetylshanzhiside, deoxypulchelloside I and 5-desoxy-lamiide are reported for the first time. Fortunately, the main IGs were detected in both the aerial parts and the underground parts of *L. rotata*; unfortunately, a few minor bases, such as schismoside and 5-desoxylamiide, were not detected in the underground parts. In general, the aerial part has a similar IGs profile to that of the underground part of *L. rotata*.

**Table 2:** The identification iridoid glycosides of *L. rotata* by UPLC-Q/TOF-MS^a^

Peak no.	RT (min)	Compound	Formula	Calculated (Da)	Selected ion	Precursor ion (Da)	Mass accuracy (ppm)	Root part	Aerial part
1	3.765	7-*epi*-Phlomiol	C_17_H_26_O_13_	438.1373	[M + Na]^+^	461.1275	0.4	✓	✓
2	4.056	Schismoside	C_17_H_26_O_12_	422.1424	[M + Na]^+^	445.1308	−3.6	—	✓
3	4.969	5-Deoxypulchelloside I	C_17_H_26_O_11_	406.1475	[M + Na]^+^	429.1360	−3.5	✓	✓
4	5.428	Phlorigidoside C.	C_17_H_24_O_11_	404.1319	[M + Na]^+^	427.1221	0.5	✓	✓
5	5.665	Loganic acid	C_16_H_24_O_10_	376.1370	[M + Na]^+^	399.1263	−1.8	✓	✓
6	6.057	Unknown	C_17_H_24_O_11_	404.1319	[M + Na]^+^	427.1209	−2.3	—	✓
7	7.092	Lamalbide	C_17_H_26_O_12_	422.1424	[M + Na]^+^	445.1325	0.2	✓	✓
8	8.599	Penstemoside	C_17_H_26_O_11_	406.1475	[M + Na]^+^	429.1402	6.3	✓	✓
9	8.802	Sesamoside	C_17_H_24_O_12_	420.1268	[M + Na]^+^	443.1158	−2.3	✓	✓
10	10.093	Unknown	C_16_H_24_O_12_	408.1267	[M + Na]^+^	431.1163	−0.9	✓	✓
11	10.127	8-*O*-Acetylshanzhiside	C_18_H_26_O_12_	434.1424	[M + Na]^+^	457.1318	−1.3	✓	✓
12	10.668	Shanzhiside methyl ester	C_17_H_26_O_11_	406.1475	[M + Na]^+^	429.1360	−3.5	✓	✓
13	10.972	10-Methylixoside	C_17_H_22_O_11_	402.1162	[M + Na]^+^	425.1046	−3.8	✓	✓
14	11.398	6-*O*-Acetylshanzhiside methyl ester	C_19_H_28_O_12_	448.1581	[M + Na]^+^	471.1484	0.6	✓	✓
15	11.736	Unknown	C_17_H_22_O_11_	402.1162	[M + Na]^+^	425.1046	−3.8	—	✓
16	11.803	Phloyoside II	C_17_H_25_ClO_12_	456.1034	[M + Na]^+^	479.0951	3.5	✓	✓
17	11.972	5-Desoxylamiide	C_17_H_26_O_11_	406.1475	[M + Na]^+^	429.1360	−3.5	—	✓
18	12.840	Chlorotuberroside	C_17_H_25_ClO_11_	440.1085	[M + Na]^+^	463.1010	5.4	✓	✓
19	13.466	7,8-Dehydropenstemo side	C_17_H_24_O_11_	404.1319	[M + Na]^+^	427.1221	0.5	✓	✓
20	13.737	7,8-Dehydropenstemonoside	C_17_H_20_O_10_	388.1369	[M + Na]^+^	411.1280	2.7	✓	✓
21	13.906	Zaluzioside	C_17_H_24_O_11_	404.1319	[M + Na]^+^	427.1221	0.5	✓	✓
22	14.703	Deoxypulchelloside I	C_17_H_26_O_11_	406.1475	[M + Na]^+^	429.1360	−3.5	✓	✓
23	15.332	Loganin	C_17_H_26_O_10_	390.1526	[M + Na]^+^	413.1412	−3.4	✓	✓
24	17.231	8-*O*-Acetylshanzhiside methyl ester	C_19_H_28_O_12_	448.1581	[M + Na]^+^	471.1484	0.6	✓	✓
25	17.772	Unknown	C_16_H_24_O_12_	408.1267	[M + Na]^+^	431.1171	0.9	✓	✓
26	18.231	7-*epi*-Loganin	C_17_H_26_O_10_	390.1526	[M + Na]^+^	413.1417	−2.2	✓	✓

a ✓ have been detected, — not have been detected.

The IGs of *L. rotata* varied in concentration. In the 5,6-, 6,8-, 7,8-di-*O*-, and 6,7,8-tri-*O*-substituted glycosides,^28^ the hydroxyl group substitutions cause CH_3_OH and/or H_2_O groups to be lost when the glycosides are fragmented in the positive ion mode. If a hydroxyl group is linked to C-6, it is easy to lose a molecule of methanol and to form a lactone with the carboxymethyl (COOCH_3_) group at the C-4 position, with a (neutral) loss of 32 Da.^29,30^ If a hydrogen rather than a hydroxyl group is linked to the C-6 position, a molecule of methanol may nonetheless be lost, with the formation of a ketone and a COOCH_3_ at the C-4 position; again, a neutral loss of 32 Da occurs.

If a hydroxyl group is linked to the 1, 5, 7, or 8 position, then a H_2_O molecule will be lost, and a double bond will be formed with an adjacent carbon atom. The ease of hydroxyl group loss is in the following order: 1-OH > 5-OH > 8-OH > 7-OH. The hydroxyl group at C-1 is linked to a vinyl ether bond and can thus readily form part of a molecule of H_2_O. The hydroxyl groups at the C-5 and C-8 position are linked to quaternary carbons; the hydroxyls at either position thus contribute more readily to the loss of an H_2_O than does the hydroxyl at C-7. Losses of H_2_O and CH_3_OH groups allow the positions of the relevant hydroxyl groups to be tentatively identified.

**Fig. 3:**
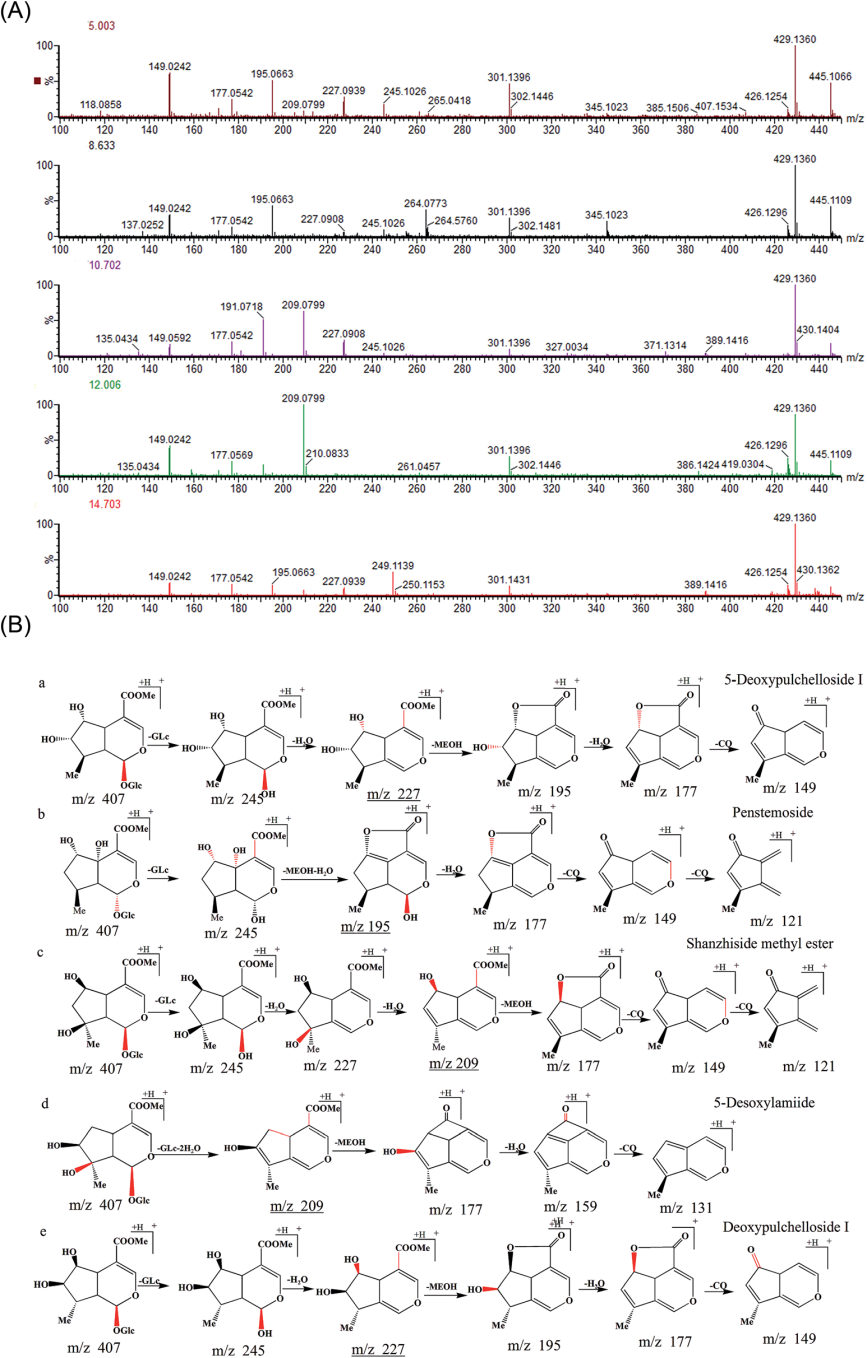
(A) MS^2^ spectra and (B) the fragmentation pathways of five compounds. (a) 5-Deoxypulchelloside I; (b) penstemoside; (c) shanzhiside methyl ester; (d) 5-desoxylamiide; (e) deoxypulchelloside I.

Numbers 3, 8, 12, 17, and 22 exhibited the same [M + Na]^+^ ions at *m*/*z* 429 and [M + K]^+^ ions at *m*/*z* 445 in the positive ion mode, consistent with a molecular formula of C_17_H_26_O_11_. Peak 12, identified by reference to the shanzhiside methyl ester standard (6,8-di-hydroxyl-substituted), exhibited [M + H]^+^ ions at *m*/*z* 407.1493 Da. Peak 12 generated [M + H-Glu]^+^at *m*/*z* 245.1026 Da, corresponding to the neutral loss of a glucose unit (Δ*m* = 162 Da), as observed in the tandem mass spectrometry (MS/MS) spectra. After successive losses of H_2_O groups (the hydroxyl groups at the C-1 and C-8 positions), ions were observed at *m*/*z* 209 Da (61% relative abundance). This observation indicated that the hydroxyl group at the C-8 position was more readily lost than the hydroxyl group at the C-6 position; the latter hydroxyl lost a molecule of methanol and formed lactones with the COOCH_3_ group at the C-4 position with neutral losses of 32 Da, yielding product ions at *m*/*z* 177 Da (32% relative abundance). However, notably, the peak yielded ions at *m*/*z* 191 (51% relative abundance, which was higher than the relative abundance of the product ion at *m*/*z* 177 Da). This difference indicated that the hydroxyl group at the C-6 position more readily formed a double bond with the group at the C-5 position than lactones with the COOCH_3_ group at the C-4 position. The product ions at *m*/*z* 149 and 121 Da were attributable to successive losses of CO groups. The proposed fragmentation pathway of the shanzhiside methyl ester is shown in Fig. 3.

Similarly, peak 3 generated ions at *m*/*z* 245, 227, 209, 195, 177 and 149 Da. The ions at *m*/*z* 245 Da corresponded to the neutral loss of a glucose unit (Δ*m* = 162 Da), and those at *m*/*z* 227 Da (Δ*m* = 18 Da) corresponded to the loss of the hydroxyl group linked to the C-1 position. With further peaks simultaneously appearing at *m*/*z* 195 Da (Δ*m* = 32 Da, the hydroxyl group linked to the C-6 position), no other hydroxyl group was linked to the C-5 or C-8 position. If another hydroxyl group were in fact so linked, the relative abundance of the ions at *m*/*z* 209 Da would be higher than that of the ions at *m*/*z* 195 Da. Notably, the peak yielded ions at *m*/*z* 195 Da (49% relative abundance, thus higher than the relative abundance of the product ions at *m*/*z* 209 Da [10%]). The peak also yielded ions at *m*/*z* 177 Da and 149 Da (Δ*m* = 28 Da, successive losses of CO groups). The fragmentation pathways obtained above further confirmed the deductions; peak 3 was identified as 5-deoxypulchelloside I.

Peak 8 generated a [M + H − Glu]^+^ ion at *m*/*z* 245.0993 Da, but this ion had a much lower relative abundance than the product ion at *m*/*z* 195 Da formed by the neutral loss of 50 Da, which suggested that a glucose unit was linked to C-1. This result also indicated that, in addition to the hydroxyl group at C-6, another hydroxyl group was linked to a quaternary carbon. This group should be in the C-5 or C-8 position. If the hydroxyl were linked to the C-8 position, the proposed fragmentation pathways of the compound would be similar to those of shanzhiside methyl ester. Therefore, the hydroxyl group was linked to the C-5 position. Based on the above evidence, the compound was tentatively identified as penstemoside.

Peak 17 yielded a [M + H–Glu − 2H_2_O]^+^ ion at *m*/*z* 209.0799 Da by losing a glucose residue (162 Da) and two H_2_O units (36 Da), and it generated ions at *m*/*z* 177, 149, and 121 Da. However, the peak also yielded ions at *m*/*z* 159 and 131 Da (Fig. 3). These peaks indicated that a hydrogen, rather than a hydroxyl group, was linked to the C-6 position. The compound lost a molecule of methanol and formed a ketone group with the COOCH_3_ at the C-4 position with neutral losses of 32 Da. As compound 17 does not have a hydroxyl group substituent at C-6, peak 17 was tentatively identified as 5-desoxylamiide (phlomoside A) by reference to the literature data on the *L. rotata* and *Lamium* species.^19,29,30^

Peak 22 had a retention time of 14.686 min on the chromatogram. The MS/MS spectra exhibited four characteristic fragment ions at *m*/*z* 147, 177, 195, and 227 Da; the fragmentation pathways of the compound were similar to those of 5-deoxypulchelloside I. Peak 22 was tentatively identified as an isomer of 5-deoxypulchelloside I by reference to the literature data on *L. rotata* and *Lamium* species. The proposed fragmentation pathways of five compounds are shown in Fig. 3. Peaks 4 (phlorigidoside C), 19 (7,8-dehydropenstemoside), and 21 (zaluzioside) exhibited the fragmentation patterns of IGs, as did peaks 2 (schismoside), 7 (lamalbide), 14 (6-*O*-acetylshanzhiside methyl ester), 24 (8-*O*-acetylshanzhiside methyl ester), 23 (loganin), and 26 (7-*epi*-loganin). Other IGs, including sesamoside, chlorotuberoside, 7-*epi*-phlomiol, and phloyoside II, were identified by reference to standards purified from *L. rotata* in our laboratory.

### 3.2. Multivariate statistical analysis

**Fig. 4:**
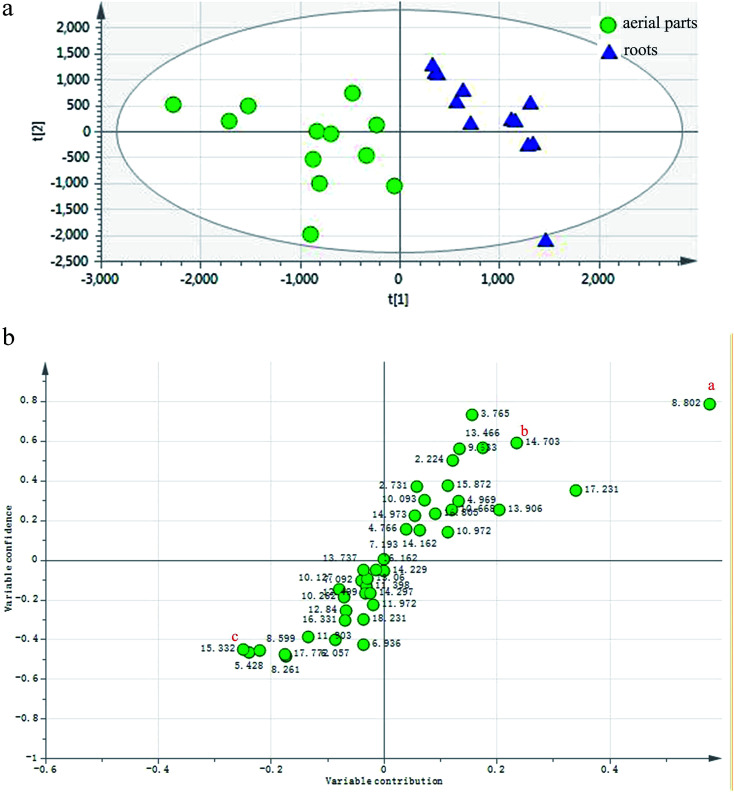
(a) PCA/Score plot of the aerial and root parts of *L. rotata*. (b) OPLS-DA/S-plot of the aerial and root parts of *L. rotata*. a: (*t*_R_ 8.80 min, *m*/*z* 443.1158).

**Fig. 5:**
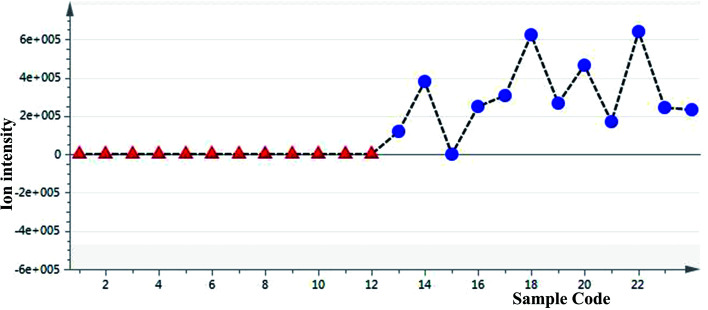
Selected ion intensity trend plots. a/sesamoside: (*t*_R_ 8.80 min, *m*/*z* 443.1158) ▲: aerial parts; ●:roots.

To define non-obvious differences between the underground and aerial parts of *L. rotata*, a UPLC-TOF-MS-based metabolomic approach was designed to investigate the chemical characteristics of the two parts of *L. rotata*. Principal component analysis (PCA) and orthogonal partial least-squares discriminant analysis (OPLS-DA) were performed. As shown in Fig. 4a, the 12 samples clustered into two groups when non-targeted PCA scores were plotted after Pareto scaling with mean centring. The chemical characteristics of the underground and aerial parts of *L. rotata* were significantly different. Extended statistical analyses were used to provide an S-plot to identify the components contributing the most to the observed differences (Fig. 4b). In this plot, each point represents an ion *t*_R_–*m*/*z* pair; the *t*_R_–*m*/*z* pair points at the two ends of the “S” are the markers affording the maximum discriminatory confidence for either group. At least three constituents (a–c) could be used to discriminate clearly between the underground and aerial parts of *L. rotata*. The ion intensity trends of these selected ions in the analysed samples are shown in Fig. 5. The component associated with ion a (*t*_R_ 8.80 min, *m*/*z* 443.1158) is the most suitable chemical marker for distinguishing the aerial and underground parts of *L. rotata*; the potential marker ion a was identified as sesamoside, which was present at low levels in the aerial parts but was abundant in the underground parts of *L. rotata*.

Although sesamoside was found to be the key factor to distinguish the two parts of the herb, it should be emphasized that 8-*O*-acetyl shanzhiside methyl ester and shanzhiside methyl ester, two major qualitative and quantitative components of *L. rotata*, contributed nothing to the observed difference between the aerial and root parts of *L. rotata* (ESI Fig. 1†). Additionally, pharmacological studies have revealed that 8-*O*-acetylshanzhiside methyl ester and shanzhiside methyl ester have haemostatic,^10,11^ analgesic,^7,8^ and anti-inflammatory bioactivities,^9^ while sesamoside possesses only weak analgesic activities.^31^ Moreover, the aerial part showed a similar IGs profile to that of the underground part of *L. rotata*. In view of the above reasons, it is still difficult to deny or support the rationality of the change in the herb part listing based only on the difference in individual ingredients between the two parts of the herb. To further explore the relationship between different parts, the total IG contents in the different samples of *L. rotata* were assayed by UV-Vis spectrophotometry.

### 3.3. Assay of total IGs and antioxidant activity in two parts of *L. rotata* samples

Twelve samples of *L. rotata*, including the aerial and underground parts, were assayed according to the method described above. The total IG contents in the different samples of *L. rotata* are shown in Table 3; the assay results showed that the total IG contents from the underground part were higher than those from the aerial parts. However, high concentrations of IG metabolites were also found in the aerial parts. In particular, the average total IG content in the aerial organs of the Gansu sample is nearly as high as that in the roots of Tibet samples. From this evidence, it can be cautiously concluded that the change in the listing of the medicinal parts of *L. rotata* is rational. The results also suggested that the dosage should be increased for therapeutic effect if the aerial part is used instead of the underground part.

**Table 3:** Contents of the total IGS and antioxidant activity in 24 samples of *L. rotata*^a^

Sample	Total IGS (mg g^−1^)	Antioxidant activity		Sample	Total IGS (mg g^−1^)	Antioxidant activity	
		DPPH (IC_50_ mg ml^−1^)	Reducing power (IC_50_ mg ml^−1^)			DPPH (IC_50_ mg ml^−1^)	Reducing power (IC_50_ mg ml^−1^)
Tibet 01A	20.03 ± 0.01	0.61 ± 0.01	33.34 ± 1.02	Tibet 01R	35.67 ± 0.10	0.58 ± 0.11	6.19 ± 0.54
Tibet 02A	19.78 ± 0.04	1.10 ± 0.14	8.26 ± 0.25	Tibet 02R	38.71 ± 0.02	0.31 ± 0.04	6.23 ± 0.35
Tibet 03A	20.09 ± 0.07	0.69 ± 0.07	5.23 ± 0.55	Tibet 03R	39.47 ± 0.07	0.55 ± 0.01	6.08 ± 0.61
Sichuan 01A	21.23 ± 0.03	1.29 ± 0.13	29.81 ± 1.33	Sichuan 01R	40.99 ± 0.15	0.37 ± 0.01	6.40 ± 0.59
Sichuan 02A	21.99 ± 0.05	1.30 ± 0.19	31.26 ± 1.39	Sichuan 02R	43.27 ± 0.07	0.37 ± 0.04	6.11 ± 0.58
Sichuan 03A	24.27 ± 0.10	1.32 ± 0.07	29.81 ± 0.92	Sichuan 03R	44.03 ± 0.01	0.21 ± 0.03	4.46 ± 0.42
Qinghai 01A	27.31 ± 0.01	0.55 ± 0.06	5.82 ± 0.48	Qinghai 01R	44.03 ± 0.11	0.06 ± 0.01	5.47 ± 0.33
Qinghai 02A	28.83 ± 0.01	0.73 ± 0.02	6.36 ± 0.41	Qinghai 02R	45.56 ± 0.03	0.28 ± 0.01	5.56 ± 0.51
Qinghai 03A	31.87 ± 0.01	0.40 ± 0.05	5.80 ± 0.39	Qinghai 03R	53.16 ± 0.01	0.25 ± 0.02	5.83 ± 0.41
Gansu 01A	32.63 ± 0.04	0.47 ± 0.07	5.67 ± 0.44	Gansu 01R	53.92 ± 0.03	0.28 ± 0.04	4.78 ± 0.23
Gansu 02A	32.63 ± 0.04	0.80 ± 0.14	5.93 ± 0.37	Gansu 02R	53.92 ± 0.08	0.24 ± 0.02	3.88 ± 0.11
Gansu 03A	33.39 ± 0.04	0.48 ± 0.05	6.20 ± 0.52	Gansu 03R	58.48 ± 0.02	0.26 ± 0.01	5.91 ± 0.47
BHT		0.01 ± 0.001	0.10 ± 0.01				

a A – the aerial parts of *L. rotata*, R – the root of *L. rotata*, each value represents the mean of three determinations (*n* = 3) and three independent experiments ± standard deviation.

To further understand the reasoning for the change in the herb part listing, the antioxidant activity was evaluated by the IC_50_ values obtained in a DPPH radical and reducing power assay. BHT was used as a positive control, for the DPPH scavenging activity and reducing power, the correlation factor (*R*) was calculated, and the values showed extremely significant correlations (*p* < 0.01). The antioxidant activity results showed a good correlation with the total IG content. Specifically, the underground part of the No. 3 sample from Sichuan had the smallest IC_50_ value of 1.06 ± 0.13 mg ml^−1^ (*p* < 0.05), indicating the highest DPPH scavenging activity. Additionally, the underground part of the No. 2 sample from Gansu had the smallest IC_50_ value of 19.41 ± 0.53 mg ml^−1^, indicating the highest reducing power (*p* < 0.05). On the other hand, the lowest DPPH scavenging activity was found in the aboveground part of the No. 3 sample from Sichuan, with an IC_50_ value of 6.58 ± 0.36 mg ml^−1^ (*p* < 0.05), and the lowest reducing power was found in the aerial part of the No. 1 sample from Tibet, with an IC_50_ value of 166.69 ± 5.08 mg ml^−1^ (*p* < 0.05).

In the presence of antioxidants, both the underground part and the aerial parts exhibited excellent antioxidant activity. The total IG content dramatically affected the antioxidant activity. The aerial parts of specific geographical origins that showed high total IG contents also showed similar antioxidant activity to that of the underground part, and these results suggested that the change in the listed medicinal parts of *L. rotata* is rational.

## 4. Conclusions

*Lamiophlomis* is a monotypic genus, represented by *L. rotata*, in the family Lamiaceae. The harvesting of the root of the herb is far in excess of acceptable levels in the high-altitude grassland ecosystem. Without other alternative, the use of the roots had to be replaced by the aerial parts to conserve natural resources. However, the IG composition of the two parts still remains poorly understood. In this paper, an LC-TOF/MS and multivariate statistical analysis method for characterizing twenty-six IGs was developed. The aerial part has a similar chemical profile to that of the underground part of *L. rotata*, although a slight difference in the IG composition of the two parts was detected. Metabolomic studies indicated that the concentration of sesamoside was higher in the roots, but the ion intensity trend plots of shanzhiside methyl ester and 8-*O*-acetylshanzhiside methyl ester did not differ significantly between the aerial parts and the roots of *L. rotata* from various areas. The total IG contents appear to be controlled more by environmental factors than by the location in different parts of the plant: the aerial parts of specific geographical origin have high total IG contents similar to those of the corresponding underground parts. Meanwhile, *in vitro* antioxidant activity was assayed to estimate the rationality of the change in medicinal parts, and the results suggested that when the underground part is replaced by the aerial part, the dosage should be increased in traditional prescriptions. Therefore, it may be reasonable to choose the aerial parts instead of the roots to protect the high-altitude grassland ecosystem. These results also contribute to the rationale for altering the herb parts used in the Chinese Pharmacopoeia.

## Conflicts of interest

The authors declare no conflicts of interest associated with this manuscript.

## Supplementary Material

RA-008-C7RA10143K-s001
