# Supplementary material for: The similarity and variability of the iridoid glycoside profile and antioxidant capacity of aerial and underground parts of Lamiophlomis rotata according to UPLC-TOF-MS and multivariate analyses
Source: RSC Adv. 2018 Jan 10;8(5):2459–68. doi: 10.1039/c7ra10143k (PMC9077588; doi:10.1039/c7ra10143k)
Supplement: RA-008-C7RA10143K-s001 [file RA-008-C7RA10143K-s001.pdf]

Electronic Supplementary Material (ESI) for RSC Advances.
This journal is © The Royal Society of Chemistry 2018

The captions of the supplementary figures

Supplementry Fig.1 Selected ion intensity trend plots. a: shanzhiside methylester: (t_R 10.67 min, *m/z* 429.1360); b: 8-*O*-shanzhiside methyl ester: (t_R 10.67 min, *m/z* 429.1360), ●: aerial parts; ▲:roots

Supplementry Fig.2 TIC chromatography (positive) of IG standards and NO.12 sample

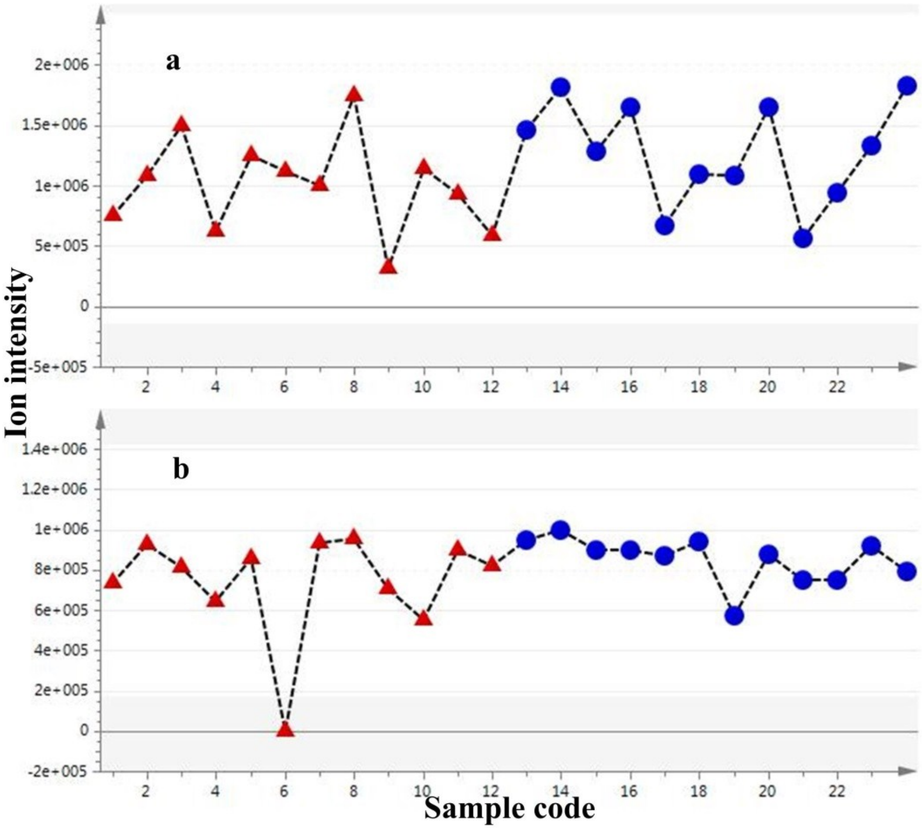

Supplementry Fig.1

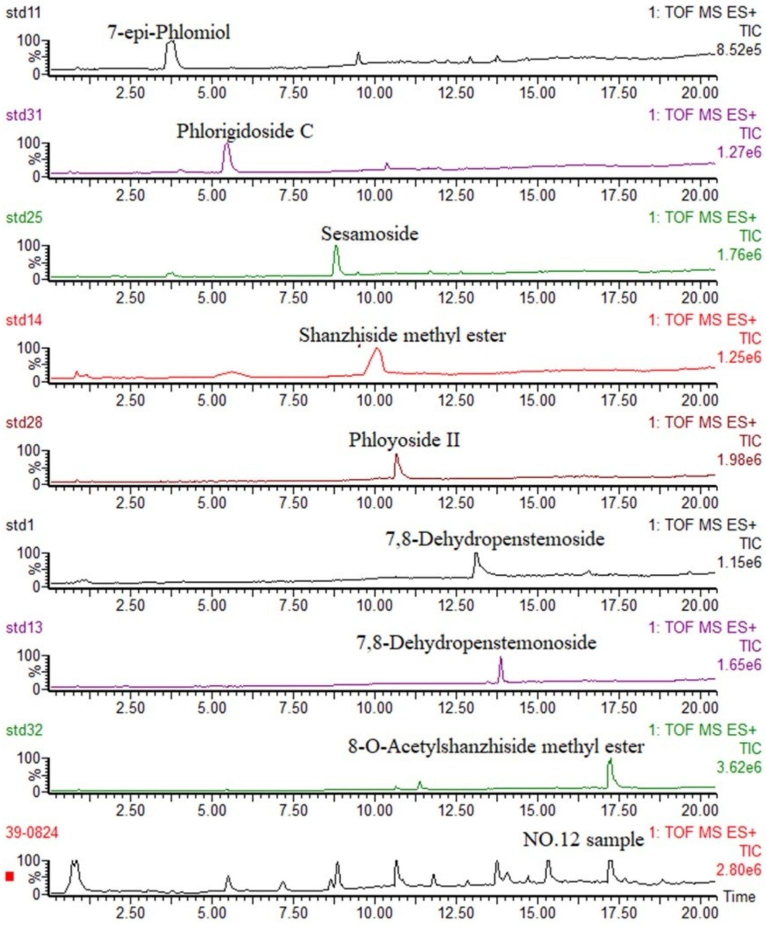

Supplementry Fig.2
